# Diastolic Dysfunction in Acute and Critical Illness: Acute Pathophysiology to Chronic Heart Failure

**DOI:** 10.1016/j.jacadv.2025.102532

**Published:** 2026-01-13

**Authors:** Tom Fisher, Marcus Abbawy, Finlay Holden, James Cotton, Sandeep Hothi, Thomas Ingram, Kesaven Dhamodaran, Suneesh Thilak, Cyril Chacko, Fang Gao-Smith, Tonny Veenith

**Affiliations:** a The Royal Wolverhampton NHS Trust, New Cross Hospital, Wolverhampton, United Kingdom; b University College London Hospitals NHS Foundation Trust, London, United Kingdom; c The Dudley Group NHS Foundation Trust, Dudley, United Kingdom; d University Hospitals Birmingham, Mindelsohn Way Edgbaston, Birmingham, United Kingdom

**Keywords:** critical care, diastolic dysfunction, ejection fraction, heart failure

## Abstract

Heart failure with preserved ejection fraction (HFpEF) is a heterogeneous, multi-organ syndrome driven by comorbidity-induced systemic inflammation. In acute and critical illness, such as sepsis, acute diastolic dysfunction is common. Its prognostic significance is debated and is complicated by hemodynamic instability and diagnostic challenges.

Survivors of acute illness face a long-term risk of major adverse cardiovascular events, yet the transition from critical illness to acquired diastolic dysfunction to chronic HFpEF remains an underexplored area requiring further research.

Recent landmark trials have established new therapeutic options for chronic HFpEF, including sodium glucose co-transporter 2 inhibitors, mineralocorticoid receptor antagonists, and glucagon-like peptide-1 receptor agonists. Here, we review the pathophysiology of HFpEF across the continuum from chronic stable HFpEF to acute decompensation, identify long-term sequelae, and highlight future advancements.

## Highlights

• Acute HFpEF is a systemic inflammatory syndrome driven by comorbidities, extending beyond the myocardium.
• In critical illness, acute diastolic dysfunction is common, but its link to mortality is complex.
• New therapies (SGLT2i, GLP-1 RAs) and AI-driven diagnostics are revolutionizing HFpEF care.

Heart failure with preserved ejection fraction (HFpEF) is a complex clinical syndrome characterized by the signs and symptoms of heart failure, a left ventricular ejection fraction (LVEF) of >50%, with objective evidence of elevated left ventricular (LV) filling pressures.^1^ HFpEF accounts for over half of all patients with heart failure and is increasing in prevalence.^2^ It has been described as an independent phenotype not crossing over into heart failure with reduced ejection fraction.^3^ This burden is driven by aging and the concurrent cardiometabolic risk factors, including obesity, hypertension, and type 2 diabetes mellitus. HFpEF is no longer viewed as a singular cardiac disease-causing diastolic dysfunction, but a heterogeneous, multi-organ systemic syndrome where the heart is a principal target of a generalized disease process.

Despite a preserved LVEF, the prognosis for HFpEF is poor, with a 21% 30-day all-cause hospital readmission rate and a 1-year mortality approaching 20% to 29%, comparable with patients with heart failure and reduced ejection fraction (HFrEF).^4^ Early perceptions of HFpEF were that it was prognostically benign when compared to HFrEF, a belief which has since been challenged by more recent data.^5^ A comprehensive meta-analysis revealed that while cardiovascular mortality remains higher in HFrEF, the all-cause mortality in HFpEF is significantly higher (47.3% vs 43.7% in HFrEF).^6^

This suggests that HFpEF is associated with a systemic process, driven largely by noncardiovascular disease, causing concurrent cardiac pathology. This highlights the challenges of treating HFpEF and the urgent requirement for research and a deeper understanding of its pathophysiology.^7^

This review aims to bridge the gap between the well-characterized pathophysiology of chronic, stable HFpEF and its presentation and consequences in acute illness. We will trace the disease from its molecular and cellular mechanisms to acute decompensated heart failure (ADHF) in the intensive care unit (ICU), explore the long-term cardiovascular risks faced by ICU survivors, and the modern diagnostic and therapeutic opportunities that offer effective treatments for this syndrome. HFpEF remains an understudied syndrome in critical care with new literature showing its increased prevalence and its effect on patient outcome.

## The systemic and multi-organ pathophysiology of HFpEF

**Figure 1:**
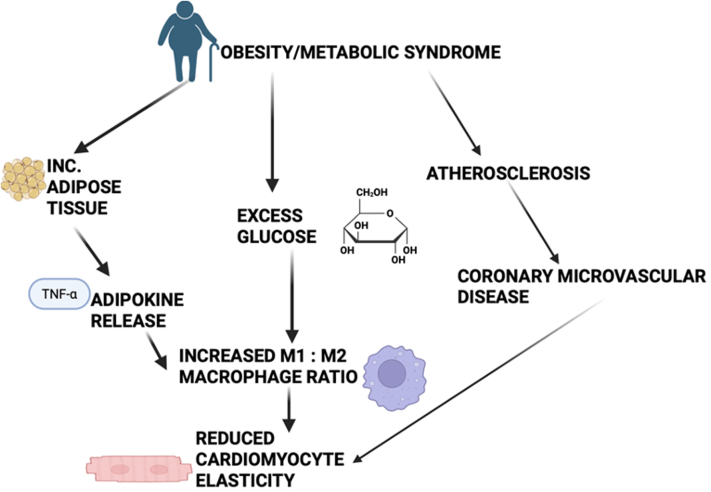
The Systemic Pathophysiology of HFpEF Comorbidities, including obesity, type 2 diabetes, hypertension, chronic kidney disease (CKD), and aging, contribute to a systemic pro-inflammatory state and endothelial dysfunction, resulting in multi-organ consequences. HFpEF = heart failure with preserved ejection fraction; TNF-α = tumour necrosis factor alpha.

HFpEF originates from a systemic, low-grade pro-inflammatory state induced by common comorbidities, such as obesity, type 2 diabetes, hypertension, chronic kidney disease, and aging (Figure 1).^3^ This state of chronic “meta-inflammation” and “inflammaging” creates a hostile systemic environment characterized by oxidative stress and endothelial dysfunction, which targets multiple organ systems. This process can be summarized as a “first hit,” creating a vulnerable environment that predisposes organs to injury. The myocardium, with its high metabolic demand and dense microvasculature, is susceptible, setting the stage for a “second hit” to translate systemic stress into myocardial dysfunction.^8^

### Myocardial remodeling: the triad of stiffness, fibrosis, and hypertrophy

The functional abnormality in HFpEF is increased LV diastolic stiffness, arising from a combination of intrinsic cardiomyocyte stiffness and interstitial fibrosis.^9^

• **Cardiomyocyte stiffness and titin:** At the cellular level, elevated cardiomyocyte stiffness is the primary driver of diastolic dysfunction, a property governed by titin, a protein that functions as a bidirectional molecular spring, contributing to passive stiffness and elastic recoil. Post-translational modifications, particularly phosphorylation, dynamically regulate titin. Systemic inflammation and coronary microvascular dysfunction (CMD) in HFpEF lead to reduced bioavailability of nitric oxide (NO) and to attenuated activity of its downstream effector, protein kinase G. This results in hypophosphorylation of regions within titin, making it stiffer, increasing cardiomyocyte resting tension, and impairing diastolic relaxation.^10^^,^^11^
• **Myocardial fibrosis and transforming growth factor-β:** This stiffness is compounded by reactive interstitial fibrosis, driven by excessive deposition of extracellular matrix proteins, such as collagen, by cardiac fibroblasts. The regulator of this process is transforming growth factor-β (TGF-β), a potent profibrotic cytokine. In the inflamed myocardial microenvironment, TGF-β is secreted by immune cells (eg, macrophages) and resident cardiac cells in response to mechanical and inflammatory stress. Activated TGF-β signals through both canonical (dependent on the SMAD family of signalling proteins) and noncanonical pathways to induce the transformation of quiescent fibroblasts into activated, collagen-secreting myofibroblasts, leading to progressive myocardial fibrosis and stiffening.^12^, ^13^, ^14^
• **Myocyte hypertrophy:** Concentric LV remodeling or hypertrophy is a frequent, though not universal, finding in HFpEF, representing an adaptive response to pressure overload from conditions like hypertension and increased LV end-diastolic pressure secondary to impaired relaxation.^15^

### Role of coronary microvascular and endothelial dysfunction

The “Paulus and Tschöpe” hypothesis provides a unifying framework linking systemic inflammation to myocardial dysfunction. It suggests that comorbidity-induced systemic inflammation is the upstream event that triggers widespread coronary microvascular endothelial dysfunction, leading to reduced NO bioavailability and initiating a cascade of titin hypophosphorylation and TGF-β activation, resulting in myocardial stiffness and fibrosis. This hypothesis demonstrates a high prevalence (nearly 75%) of CMD in patients with HFpEF, which correlates directly with disease severity, natriuretic peptide levels, and right ventricular dysfunction.^16^ Clinical evidence now confirms that microvascular dysfunction is a dominant feature of the HFpEF syndrome. A 2024 systematic review and meta-analysis established the pooled prevalence of any type of CMD at 58% (95% CI: 42% to 74%). The pooled analysis for clinical outcomes revealed that, in patients with HFpEF, the presence of CMD is associated with an HR of 3.19 (95% CI: 1.04-9.57; *P* = 0.04) for the composite endpoint of death or hospitalization for heart failure.^17^ Despite recruiting 14 observational studies enrolling a total of 1,138 patients with HFpEF, this study has limitations as 5 of these observational studies were retrospective, and the pooled analysis’s reliability is reduced due to the variability in CMD and HFpEF diagnosis definitions.

### Immune cell activation in the myocardium

Histopathology reveals infiltration of inflammatory cells, with a particular shift in the macrophage population from protective, anti-inflammatory resident macrophages to pro-inflammatory, monocyte-derived macrophages (CCR2+).^18^ These activated immune cells perpetuate local inflammation and fibrosis. A key intracellular signaling hub for this process is the NOD (nucleotide-binding oligomerization domain)-like receptor protein 3 (NLRP3) inflammasome, a multiprotein complex within cardiomyocytes and fibroblasts. When activated by cellular stress signals (damage-associated molecular patterns) common in HFpEF, the NLRP3 inflammasome triggers the maturation and release of potent pro-inflammatory cytokines, interleukin-1β, and interleukin-18, which drive local inflammation, fibrosis, and a form of inflammatory cell death known as pyroptosis.^19^^,^^20^

### Extracardiac manifestations: the cardiorenal-metabolic axis

HFpEF is a multi-organ syndrome, with the interplay between the heart and kidneys being particularly crucial. Cardiorenal syndrome in HFpEF is a bidirectional process. Elevated right-sided cardiac pressures lead to renal venous congestion, which can cause renal interstitial fibrosis and worsen kidney function. Conversely, primary chronic kidney disease, a common comorbidity, contributes to systemic inflammation, neurohormonal activation (renin-angiotensin-aldosterone system), and volume overload, thereby exacerbating cardiac dysfunction.^21^ Beyond the kidneys, dysfunction extends to skeletal muscle, where impaired oxygen extraction contributes to exercise intolerance, and to adipose tissue, which functions as a pro-inflammatory endocrine organ, secreting adipokines that fuel the systemic inflammatory state.

## Diastolic dysfunction in critical illness

### Acute decompensated heart failure

Patients with HFpEF account for 40% to 50% of ADHF presentations. Clinically, they differ from patients with HFrEF, as they are older, more often female, and frequently present with hypertension rather than hypotension.^22^ The stiff, noncompliant LV in HFpEF has limited preload reserve, making these patients exquisitely sensitive to fluctuations in volume status and heart rate. Tachyarrhythmias, such as atrial fibrillation, precipitate cardiogenic shock.^8^^,^^23^ Despite these physiological differences, postdischarge outcomes following an ADHF admission are similarly poor for both HFpEF and HFrEF, underscoring the severity of the decompensated state regardless of baseline LVEF.^22^

### Sepsis-associated myocardial dysfunction

In the ICU, sepsis is a potent trigger for acute cardiac dysfunction, a condition termed “septic cardiomyopathy.”^24^ While systolic dysfunction has long been recognized, recent evidence highlights the impact of sepsis on diastolic function. In a 2025 large contemporary multicenter observational study that involved echocardiography assessment in 402 patients at 4 different occasions during ICU stay, as per American-European guidelines, acute LV diastolic dysfunction, which differs to acute HFpEF, was observed in up to 76% of patients with septic shock.^25^ The pathophysiology is multifactorial, driven by the septic cascade itself: a storm of inflammatory cytokines, widespread activation of inducible NO synthase, and sympathetic hyperactivity converge to impair myocardial relaxation, disrupt intracellular calcium handling, and induce mitochondrial dysfunction.^26^

### Prognostic significance in sepsis

The prognostic importance of acute diastolic dysfunction in sepsis has been a subject of considerable debate. While smaller, earlier studies reported an association between diastolic dysfunction and increased mortality, this finding has not been consistent.^27^ The most robust and current evidence comes from the prospective, multicenter PRODIASYS (Prognostic Assessment of Diastolic and Systolic Left Ventricular Function in Septic Shock) study, which focuses on the acute condition sepsis, found that despite its high prevalence (76%), the presence of diastolic dysfunction was not independently associated with 28-day mortality, suggesting that acute, reversible diastolic dysfunction may be a common response to the profound physiological stress of sepsis, rather than a specific marker of underlying cardiac vulnerability that independently drives mortality.^25^ Myocardial work indices, such as the global work index, being used as a prognostic marker in sepsis, are increasing in prevalence and could have a significant role in the future of measuring sepsis prognosis.

However, this does not mean that cardiac function is irrelevant to outcomes, but specific quantitative measures of hemodynamic compromise appear to be more prognostically valuable than a binary diagnosis of LV diastolic dysfunction.^28^ An elevated E/e' ratio, which reflects high LV filling pressures, has been more consistently linked to adverse outcomes in some cohorts. Furthermore, impaired global longitudinal strain, a more sensitive marker of intrinsic myocardial contractility than LVEF, is emerging as a powerful and independent predictor of mortality in septic shock.^29^^,^^30^

### Diagnostic challenges in the ICU

Assessing diastolic function at the bedside of a critically ill patient is challenging. Standard echocardiographic parameters are heavily influenced by the rapidly changing physiology of the ICU environment.^28^ Positive pressure ventilation alters intrathoracic pressures and cardiac loading conditions. Vasoactive medications directly affect preload, afterload, and heart rate.^28^ Large-volume fluid resuscitation and ongoing capillary leak cause dynamic shifts in volume status. Consequently, many traditional diastolic parameters lose their validity.^28^ For instance, the E/e' ratio is better interpreted as a surrogate for left atrial pressure rather than a pure measure of intrinsic diastolic function in this context. Among the available metrics, the tissue Doppler velocity of the lateral mitral annulus (lateral e') is considered a less load-dependent measure, and it has demonstrated the strongest correlation with mortality in critically ill populations.^28^ The echocardiographic indices used for diagnosis have not been validated in patients on invasive ventilation or receiving vasopressors. Although the incidence of HFpEF in critical care patients is higher than in the general population, the validity of the diagnostic imaging should be correlated with hemodynamic parameters.

This concept illustrates the potential trajectories of patients following ICU admission. On the left, the continuum begins with an acute insult (such as sepsis), which triggers a cytokine storm, vasoplegia, and mitochondrial dysfunction—culminating in acute diastolic dysfunction during critical illness. Recovery occurs in approximately 33% of survivors, marked by a return to normal diastolic function. However, around 67% of survivors experience persistent diastolic dysfunction, an increased long-term risk of major adverse cardiovascular events (MACE).^25^ This trajectory ultimately leads to a state of chronic HFpEF, characterised by established myocardial fibrosis and structural remodelling.^12^

## Long-term cardiovascular sequelae of acute and critical illness

### Postsepsis syndrome and heightened cardiovascular risk

Survival from critical illness, particularly sepsis, is often not a return to baseline health. Many survivors experience long-term impairments known as postintensive care syndrome, and a key component of this is a markedly elevated risk for future cardiovascular events.^26^^,^^31^ Epidemiological studies have consistently shown that sepsis survivors face a long-term risk of developing heart failure, myocardial infarction, and stroke.^32^ Heart failure is the second most common reason for hospital readmission following a septic episode, and a history of sepsis is now considered a nontraditional risk factor for the development of cardiovascular disease.^24^^,^^33^

### Acute dysfunction to chronic HFpEF

**Figure 2:**
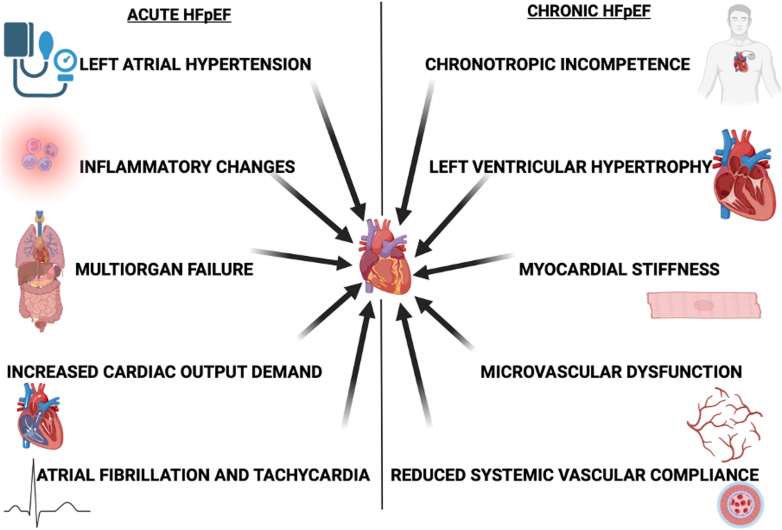
The Continuum of Diastolic Dysfunction: From Critical Illness to Chronic HFpEF This concept illustrates the potential trajectories of patients following ICU admission. On the left, the continuum begins with an acute insult (such as sepsis), which triggers a cytokine storm, vasoplegia, and mitochondrial dysfunction—culminating in acute diastolic dysfunction during critical illness. Recovery occurs in approximately 33% of survivors, marked by a return to normal diastolic function. However, around 67% of survivors experience persistent diastolic dysfunction, progressing toward postsepsis syndrome and an increased long-term risk of major adverse cardiovascular events (MACE).^25^ This trajectory ultimately leads to a state of chronic HFpEF, characterized by established myocardial fibrosis and structural remodeling.^12^ ICU = intensive care unit; other abbreviation as in Figure 1.

This increased risk questions the long-term fate of the diastolic dysfunction that develops during critical illness. This represents a significant knowledge gap in both cardiology and critical care. The PRODIASYS study provided a vital clue, while diastolic function normalized in one-third of sepsis survivors, it remained abnormal in the majority.^25^ This large cohort of patients with persistent, postsepsis diastolic dysfunction may represent a population in transition, actively progressing toward a clinical diagnosis of chronic HFpEF. Figure 2 highlights the potential acute and chronic trajectories following critical illness and ICU admission.

A central challenge is differentiating between pre-existing subclinical diastolic dysfunction that is “unmasked” by the stress of critical illness and de novo myocardial injury and remodeling caused by the acute disease. Critical illness may act as a physiological “stress test.” In individuals with pre-existing risk factors and a vulnerable myocardial substrate, the massive inflammatory and hemodynamic insult of sepsis may accelerate the underlying disease process, transforming subclinical dysfunction into a persistent and clinically significant state.^34^ Therefore, the development of diastolic dysfunction in the ICU may not be a transient epiphenomenon but rather a sentinel event that identifies a high-risk population requiring targeted cardiological surveillance and intervention after hospital discharge.^32^

### Postintensive care syndrome and cardiovascular health

The long-term impact of critical illness extends beyond organ-specific pathology. Postintensive care syndrome (PICS) describes the new or worsening impairments in physical, cognitive, and psychological health that persist long after discharge, interconnected with cardiovascular health. The profound muscle weakness and fatigue (ICU-acquired weakness) characteristic of PICS can severely limit a patient's ability to engage in physical activity or participate in cardiac rehabilitation for cardiovascular risk reduction.^35^ Furthermore, the high prevalence of anxiety, depression, and post-traumatic stress disorder in PICS is not only a significant source of morbidity but also an independent risk factor for adverse cardiovascular events.^36^

## Diagnosis and management

The management of HFpEF was limited to diuretics for symptom control and treatment of comorbidities. However, over the last 5 years, a therapeutic evolution has occurred, with multiple landmark trials establishing new pillars of evidence-based therapy.

### Therapies for chronic HFpEF

A summary of the landmark clinical trials that have reshaped the therapeutic landscape for HFpEF is presented in Table 1.

**Table 1:** Landmark Clinical Trials of Medical Therapies in HFpEF

Trial Name (Year)	Drug Class	Drug	N	Key Population	Primary Endpoint	Results (HR/RR) 95% CI; *P* Value
EMPEROR-Preserved (2021)^37^	SGLT2i	Empagliflozin	5,988	LVEF >40%	CV death or HF hospitalization	HR 0.79 (0.69-0.90); *P* < 0.001
DELIVER (2022)^37^	SGLT2i	Dapagliflozin	6,263	LVEF >40%	CV death or worsening HF	HR 0.82 (0.73-0.92); *P* < 0.001
TOPCAT (2014)^38^	MRA	Spironolactone	3,445	LVEF ≥45%	CV death, aborted cardiac arrest, or HF hospitalization	HR 0.89 (0.77-1.04); *P* = 0.14
FINEARTS-HF (2024)^4^	MRA	Finerenone	>7,400	LVEF ≥40%	CV death or HF events	Significant reduction
PARAGON-HF (2019)^39^	ARNI	Sacubitril/Valsartan	4,822	LVEF ≥45%	Total HF Hospitalizations and CV Death	RR 0.87 (0.75-1.01); *P* = 0.06
STEP-HFpEF (2023)^40^	GLP-1 RA	Semaglutide	529	Obesity, LVEF ≥45%, No diabetes	Change in KCCQ-CSS and body weight	ΔKCCQ: +16.6 + 8.7; ΔWeight: 13.3% vs −2.6% (*P* < 0.001) for bot

ARNI = angiotensin receptor-neprilysin inhibitor; CV = cardiovascular; DELIVER = Dapagliflozin Evaluation to Improve the LIVEs of Patients With PReserved Ejection Fraction Heart Failure; FINEARTS-HF = Study to Evaluate the Efficacy (Effect on Disease) and Safety of Finerenone in Participants With Heart Failure and Left Ventricular Ejection Fraction (Proportion of Blood Expelled Per Heart Stroke) Greater or Equal to 40%; GLP-1 RA = glucagon-like peptide-1 receptor agonist; EMPEROR-Preserved = EMPagliflozin outcomE tRial in Patients With chrOnic heaRt Failure With Preserved Ejection Fraction; HF = heart failure; HFpEF = heart failure with preserved ejection fraction; KCCQ-CSS = Kansas city cardiomyopathy questionnaire clinical summary score; LVEF = left ventricular ejection fraction; MRA = mineralocorticoid receptor antagonist; PARAGON-HF = Efficacy and Safety of LCZ696 Compared to Valsartan, on Morbidity and Mortality in Heart Failure Patients With Preserved Ejection Fraction; RR = rate ratio; SGLT2i = sodium-glucose cotransporter 2 inhibitor; STEP-HFpEF = Research Study to Investigate How Well Semaglutide Works in People Living With Heart Failure and Obesity; TOPCAT = Aldosterone Antagonist Therapy for Adults With Heart Failure and Preserved Systolic Function.

#### Sodium glucose co-transporter 2 inhibitors

**Figure 3:**
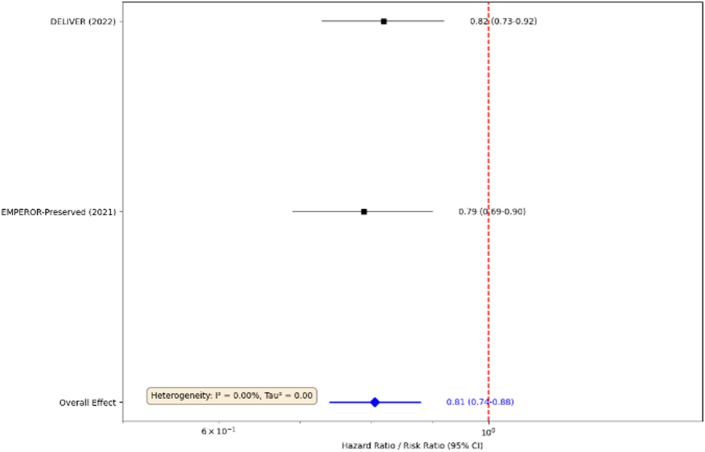
Meta-analysis of the Clinical Effect of SGLT2is in the Treatment of HFpEF Figure from Agular-Neves et al.^37^ SGLT2i = sodium-glucose cotransporter 2 inhibitor; other abbreviation as in Figure 1.

The sodium-glucose cotransporter 2 inhibitors (SGLT2is), which modulate TGF-B, oxidative stress, and inflammatory pathways, leading to reduced stiffness through the myocardial remodeling triad discussed previously, represent the first drug class to demonstrate unequivocal benefit in a broad population of patients with HFpEF.^37^ The landmark EMPEROR-Preserved (EMPagliflozin outcomE tRial in Patients With chrOnic heaRt Failure With Preserved Ejection Fraction) and DELIVER (Dapagliflozin Evaluation to Improve the LIVEs of Patients With PReserved Ejection Fraction Heart Failure) trials randomized over 12,000 patients with HFpEF (LVEF >40%) to an SGLT2i or placebo. Figure 3 illustrates the results of these trials. Both trials met their primary composite endpoint, demonstrating a significant 21% and 18% relative risk reduction, respectively, in cardiovascular death or hospitalization for heart failure. This benefit was consistent across a wide range of subgroups, including patients with and without diabetes, and was driven primarily by a robust reduction in heart failure hospitalizations. The cardioprotective mechanisms of SGLT2i are pleiotropic and extend far beyond their glucosuria, including modest diuretic and natriuretic actions, favorable shifts in cardiac metabolism toward more efficient ketone body utilization, and potent anti-inflammatory and antifibrotic effects.^37^ Based on this compelling evidence, SGLT2i have received a Class 2a (“should be considered”) recommendation in the 2022 ACC (American College of Cardiology)/AHA (American Heart Association)/HFSA (Heart Failure Society of America) heart failure guidelines.

#### Mineralocorticoid receptor antagonists

The role of mineralocorticoid receptor antagonists (MRAs) in HFpEF has been more complex. The TOPCAT (Aldosterone Antagonist Therapy for Adults With Heart Failure and Preserved Systolic Function) trial, which studied spironolactone, failed to meet its primary endpoint in the overall population.^41^ However, a prespecified geographic analysis revealed a significant benefit in patients enrolled in the Americas, in contrast to a neutral result in Russia and Georgia, raising questions about regional differences in the patient population or study conduct.^42^ More recently, the FINEARTS-HF (Study to Evaluate the Efficacy [Effect on Disease] and Safety of Finerenone in Participants With Heart Failure and Left Ventricular Ejection Fraction [Proportion of Blood Expelled Per Heart Stroke] Greater or Equal to 40%) trial investigated finerenone, a novel nonsteroidal MRA with a more favorable risk profile for hyperkalemia. This trial demonstrated a significant reduction in cardiovascular death and heart failure events. A meta-analysis combining these trials confirmed that MRAs reduce heart failure hospitalizations in patients with HFpEF, though a benefit on cardiovascular mortality was not observed.^41^ MRAs currently hold a Class 2b (“may be considered”) recommendation.

#### Angiotensin receptor-neprilysin inhibitors

The PARAGON-HF (Efficacy and Safety of LCZ696 Compared to Valsartan, on Morbidity and Mortality in Heart Failure Patients With Preserved Ejection Fraction) trial evaluated the angiotensin receptor-neprilysin inhibitor sacubitril/valsartan against valsartan alone in patients with HFpEF. The trial narrowly missed statistical significance for its primary endpoint of total heart failure hospitalizations and cardiovascular death (rate ratio: 0.87; 95% CI: 0.75-1.01; *P* = 0.06).

However, prespecified subgroup analyses suggested a potential benefit in patients with an LVEF in the lower range of preserved (45% to 57%) and in women.^39^ Based on these findings, angiotensin receptor-neprilysin inhibitors also have a Class 2b recommendation, particularly for patients with LVEF on the lower end of the HFpEF spectrum.

#### Glucagon-like peptide-1 receptor agonists

Recognizing that obesity is a major driver and a distinct phenotype of HFpEF, recent trials have targeted weight loss as a therapeutic strategy.^43^ The STEP-HFpEF (Research Study to Investigate How Well Semaglutide Works in People Living With Heart Failure and Obesity) and STEP-HFpEF-DM (Research Study to Look at How Well Semaglutide Works in People Living With Heart Failure, Obesity and Type 2 Diabetes) trials investigated the glucagon-like peptide-1 receptor agonist (GLP-1 RA) semaglutide in patients with obesity-related HFpEF, with and without diabetes. While not powered for hard clinical endpoints, both trials demonstrated profound benefits on patient-centered outcomes.^44^ Treatment with semaglutide led to substantial weight loss and significant improvements in heart failure symptoms (measured by the Kansas City Cardiomyopathy Questionnaire) and physical function (measured by the 6-minute walk distance).^43^ The mechanisms are thought to involve not only weight reduction but also direct anti-inflammatory and antifibrotic effects, positioning GLP-1 RAs as a key therapy for this highly prevalent HFpEF phenotype.^44^

### Management in the acute setting

The management of ADHF in patients with HFpEF requires a multimodal approach. While diuretics are essential for relieving congestion, the stiff, preload-dependent ventricle is susceptible to excessive volume removal, which can lead to a sharp decline in stroke volume and blood pressure. Similarly, aggressive afterload reduction with vasodilators can be poorly tolerated. Currently, there are no specific evidence-based therapies for acute diastolic dysfunction in sepsis beyond supportive care and management of the underlying infection. However, the central role of inflammation and endothelial dysfunction in both sepsis and HFpEF suggests a theoretical basis for investigating therapies that modulate these pathways in the acute setting.^45^

Acutely provoked HFpEF management centers around timely decongestion and the treatment of any precipitants. Vasodilators are often useful when approaching hypertensive presentations, while inotropes are typically reserved for shock. There is accumulating evidence regarding disease-modifying agents such as SGLT2i which are increasingly considered for early initiation. In contrast, the use of GLP-1 RAs and tirzepatide remains investigational for HFpEF.^46^

### Emerging technologies

#### Artificial intelligence in echocardiography

The diagnosis of HFpEF remains challenging, relying on the integration of clinical, biomarker, and imaging data.^47^ Although a study by Stein et al found a high correlation (*P* < 0.001) between noninvasive and invasive hemodynamic values, standardized diagnostic algorithms, such as the HFA-PEFF (Heart Failure Association Pretest Probability of Heart Failure and Preserved Ejection Fraction) and H2FPEF (Heavy, Hypertension, Atrial Fibrillation, Pulmonary Hypertension, Elder and Filling Pressures) scores, frequently yield indeterminate results, necessitating further, often invasive, testing.^48^^,^^49^ Artificial intelligence (AI) and deep learning are poised to revolutionize this process. Novel AI models have been developed that can analyze a single, standard echocardiographic video (apical 4-chamber view) and detect the subtle phenotypic signatures of HFpEF with remarkable accuracy (area under the receiver operating characteristic curve >0.9 in some validation studies).^50^ These models can accurately reclassify most cases previously deemed “indeterminate” by conventional scores and provide independent prognostic information on long-term mortality, holding promise for a faster, more accurate, and more accessible diagnostic pathway.^50^

#### Phenotyping for precision medicine

The heterogeneity of HFpEF is its defining challenge. A “one-size-fits-all” therapeutic approach is unlikely to succeed. The future of HFpEF management lies in precision medicine, which utilizes a combination of deep clinical phenotyping, advanced imaging, and multimarker biomarker strategies (including novel markers of inflammation such as growth differentiation factor 15 and fibrosis like galectin-3) to dissect the syndrome into distinct pathophysiological endotypes. Identifying these endotypes, such as the obesity-metabolic, hypertensive-stiff ventricle, or atrial fibrillation-dominant phenotypes, will enable the targeted application of therapies most likely to be effective for a given patient's underlying disease driver.

#### Targeting novel pathways

With insights into molecular pathophysiology, novel therapeutic targets are emerging. These can have positive clinical outcomes in ICU, which development ongoing in agents that directly target the core mechanisms of inflammation and fibrosis. These include direct inhibitors of the NLRP3 inflammasome (eg, MCC950), which have shown promise in preclinical models of HFpEF,^20^ and novel antifibrotic agents like relaxin, which may directly modulate extracellular matrix remodeling.^51^

#### Defining and treating ICU-acquired diastolic dysfunction

The most significant gap identified in this review is the long-term trajectory of patients who develop diastolic dysfunction in the ICU. There is an urgent need for prospective, long-term observational studies of ICU survivors with well-characterized cardiac function at the time of discharge. Such studies are essential for quantifying the absolute risk of progression to chronic HFpEF and for identifying clinical and biomarker predictors of adverse myocardial remodeling. Ultimately, this will pave the way for interventional trials testing strategies, with patients recruited from the PICS cohort. Further studies are required to delineate the treatments, such as early initiation of SGLT2i or intensive risk factor modification, in the post-ICU period, to promote myocardial recovery and prevent the transition to irreversible heart failure.

## Conclusions

The understanding of HFpEF has matured from a simple model of diastolic dysfunction to the recognition of a complex, systemic inflammatory syndrome driven by comorbidities (Central Illustration). In the crucible of critical illness, acute diastolic dysfunction is a common manifestation of this systemic stress, but its long-term consequences remain a crucial and underexplored frontier. The recent and rapid emergence of multiple practical drug classes for chronic HFpEF has transformed the therapeutic landscape, offering new hope for a condition long considered intractable. The concurrent development of powerful AI-driven diagnostic tools promises to identify patients earlier and more accurately. The next great challenge is to bridge the gap between the acute and chronic settings—to understand the transition from ICU-acquired dysfunction to established HFpEF and to translate the therapeutic breakthroughs achieved in chronic disease to the acutely ill patient and the critical illness survivor.

**Central Illustration:**
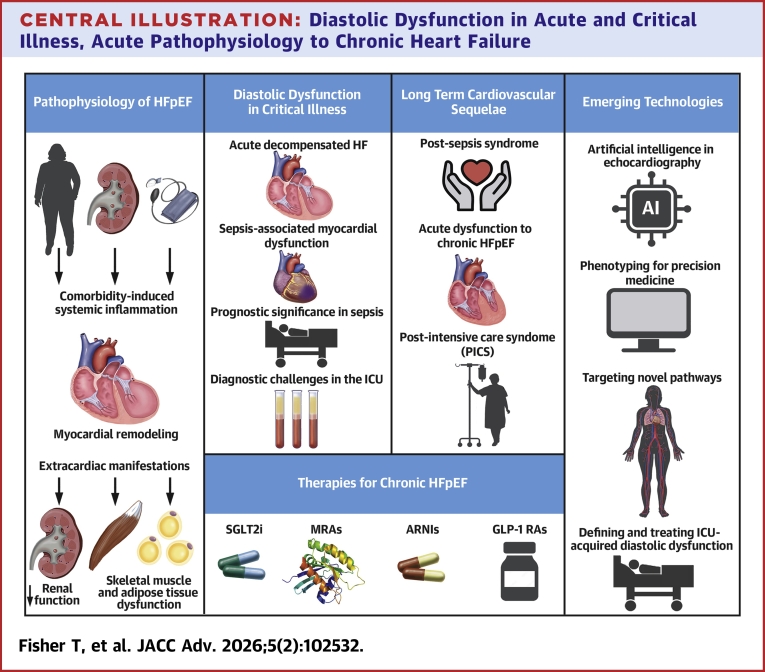
Diastolic Dysfunction in Acute and Critical Illness, Acute Pathophysiology to Chronic Heart Failure This central illustration highlights the key points of our paper. Beginning with an illustration that describes the pathophysiology of HFpEF through a display of the interplay of factors that lead to systemic inflammation and the downstream effects of this. This central illustration also highlights the diastolic dysfunction in critical illness and the long-term cardiovascular sequelae, as discussed in the study. The illustration outlines the current therapies for chronic HFpEF discussed in the study, alongside emerging technologies. AI = artificial intelligence; ARNI = angiotensin receptor-neprilysin inhibitor; GLP-1 RA = glucagon-like peptide-1 receptor agonist; MRA = mineralocorticoid receptor antagonist; SGLT2i = sodium-glucose cotransporter 2 inhibitor; other abbreviations as in Figures 1 and 2.

## Funding support and author disclosures

The authors have reported that they have no relationships relevant to the contents of this paper to disclose.
